# Diversity and evolution of the MHC class II *DRB* gene in the *Capra sibirica* experienced a demographic fluctuation in China

**DOI:** 10.1038/s41598-023-46717-5

**Published:** 2023-11-07

**Authors:** Pei-Pei Dong, Rui-Rui Wang, Shamshidin Abduriyim

**Affiliations:** 1 College of Life Science, Shihezi University, Shihezi, 832003 Xinjiang China; 2 Xinjiang Production and Construction Corps Key Laboratory of Oasis Town and Mountain-Basin System Ecology, Shihezi University, Shihezi, 832003 Xinjiang China

**Keywords:** Zoology, Genetic markers, Population genetics

## Abstract

The major histocompatibility complex (MHC) genes are the most polymorphic genes in vertebrates, and their proteins play a critical role in adaptive immunity for defense against a variety of pathogens. MHC diversity was lost in many species after experiencing a decline in size. To understand the variation and evolution of MHC genes in the Siberian ibex, *Capra sibirica*, which has undergone a population decline, we analyzed the variation of the second exon of MHC class II *DRB* genes in samples collected from five geographic localities in Xinjiang, China, that belong to three diverged mitochondrial clades. Consequently, we identified a total of 26 putative functional alleles (PFAs) with 260 bp in length from 43 individuals, and found one (for 27 individuals) to three (for 5 individuals) PFAs per individual, indicating the presence of one or two *DRB* loci per haploid genome. The Casi-DRB1*16 was the most frequently occurring PFA, Casi-DRB1*22 was found in only seven individuals, 14 PFAs occurred once, 7 PFAs twice, implying high frequency of rare PFAs. Interestingly, more than half (15) of the PFAs were specific to clade I, only two and three PFAs were specific to clades II and III, respectively. So, we assume that the polygamy and sexual segregation nature of this species likely contributed to the allelic diversity of *DRB* genes. Genetic diversity indices showed that PFAs of clade II were lower in nucleotide, amino acid, and supertype diversity compared to those of the other two clades. The pattern of allele sharing and *F*_ST_ values between the three clades was to some extent in agreement with the pattern observed in mitochondrial DNA divergence. In addition, recombination analyses revealed no evidence for significant signatures of recombination events. Alleles shared by clades III and the other two clades diverged 6 million years ago, and systematic neighbor grids showed Trans-species polymorphism. Together with the PAML and MEME analyses, the results indicated that the *DRB* gene in *C. sibirica* evolved under balancing and positive selection. However, by comparison, it can be clearly seen that different populations were under different selective pressures. Our results are valuable in understanding the diversity and evolution of the *DRB* gene in a mountain living *C. sibirica* and in making decisions on future long-term protection strategies.

## Introduction

The way a species interacts with other species or the environment is related to its genetic diversity. The ability of a species to adapt to human and natural environmental disturbances also depends on variation. The degree of genetic diversity within a species influences the ability of that species to adapt to environmental change^1^. The measurement of these variation, however, cannot be limited to the use of traditional neutral markers. The polymorphisms of Major Histocompatibility Complex (MHC) genes are considered to affect the functional plasticity of immune responses to diverse pathogenic stressors, making them excellent candidates to research adaptive evolutionary processes in natural populations^2,3^. This trait highlights the sensitivity of the immune system to environmental stresses and its importance in elucidating the mechanisms of adaptive genetic variation required for the long-term survival of a species or population^4,5^.

The most polymorphic region of the vertebrate genome that evolved under positive and balancing selection is the MHC^5–7^, a multigene family of the vertebrate adaptive immune system that contains highly polymorphic motifs that are strongly associated with immune response and disease resistance^8,9^. MHC genes belong to two main subfamilies, class I and class II, and encode proteins that are necessary for pathogen recognition and presentation to T cells. Functional class II proteins are heterodimers consisting of α and β chains, and DR subclasses are encoded by the *DRA* and *DRB* genes, respectively^10^. The amino acid residues that bind directly to the antigen are called antigen binding sites (ABS). ABSs were located at the α1 domain of α1 chain and the β1 domain of β chain, in which MHC polymorphisms in vertebrates mainly occur^11,12^.

Alpine ungulates play a significant role in maintenance the structure of vegetation and the cycling of nutrients in high-mountain ecosystems, as well as a significant source of food for predators^13,14^. However, due to its slow growth, poor rate of reproduction, vulnerability to exploitation by humans, loss of habitat, susceptibility to infectious diseases, and other reasons^15–17^, most of them are extremely vulnerable to extinction. Of these, a typical alpine hoofed species of the subfamily Caprinae (family Bovidae), is the Siberian ibex, *Capra sibirica*. This species widely habituated in the alpine regions of Central Asia, from northern India through Pakistan and Afghanistan to Russia (Siberia), and eastward to northwest China and western Mongolia^18^. According to studies, the Siberian ibex, to some extent, suffered threats from various pathogens (lethal bacteria and viruses), endoparasites (helminths) and ectoparasites (mites)^19–23^. Moreover, it shares more than 76 percent of its food with domestic animals in Chinese territory^24^, indicating not only fierce food competition but also a greatly increased risk of becoming infected. Despite the importance of MHC genes for immunological fitness, an assessment of the diversity and occurrence of these genes is still lacking in the Siberian ibex, the globally ‘Near Threatened’ mammal in Central Asia, and locally urgently needs an effective conservation and management programs^18^.

Because of anthropogenic impacts, Siberian ibex populations dropped globally and their range shrank drastically in the 1970s^25,26^. In China, particularly, it had been listed as an endangered species and given Class I protection priority in 1998^27^. Since then, Chinese scholars have started to pay attention and carry out field studies on population size and density estimation in several restricted regions in different years. For example, 593 individuals were estimated in Tashkurgan county in 2009^28^, 5604 individuals in Bay county in 2010^29^; the population density was estimated to be 2.69 individuals/km^2^ in Tomur National Natural Reserve in 2005^30^, 1.27 individuals/km^2^ in Kudi village in Kagilik county in 2010^31^ and 0–0.25 individuals/km^2^ in Tashkurgan county in 2012^32^. Later, to the best of our knowledge, there was no single report of population size and density publicly available. However, it was suggested that the population has fortunately started to recover owing to effective conservation and management (through the creation of protected areas, etc.) by the Chinese government in recent years, and thus its protection priority was decreased to Class II in 2021^33^. Generally, reduced genetic diversity is associated with demographic perturbation. Natural populations of many species that underwent a reduction in size exhibited very limited MHC diversity^34–36^. However, both theoretical and empirical studies also showed that a longer timescale of selection maintained higher MHC diversity in a population experienced demographic fluctuations^37,38^. It is thus significant to study the MHC diversity of a highly genetically diverged Siberian ibex populations^39^ during a more than half-century period of recovery.

Therefore, our objectives in this study had three facets. To begin with, we aim to comparatively evaluate the MHC diversity in different Siberian ibex populations in Xinjiang, China, and discuss our results with other species that have experienced bottlenecks. In addition, we also try to ascertain if the MHC *DRB1* divergence in different populations was in accordance with the results of mitochondrial genes divergence we reported previously^39^. Finally, to check if the MHC *DRB1* genes in the Siberian ibex that went through population fluctuation resemble the common characteristics of MHC in other vertebrates, such as positive selection, recombination, and trans-species polymorphism, and to clarify the genetic relationships of MHC *DRB1* alleles of the relic species Siberian ibex and its congenerics, including domestic goats. Our results were of importance in understanding the adaptive ability of this species and planning scientific conservation strategies to ensure long-term population development.

## Materials and methods

### Samples

**Figure 1:**
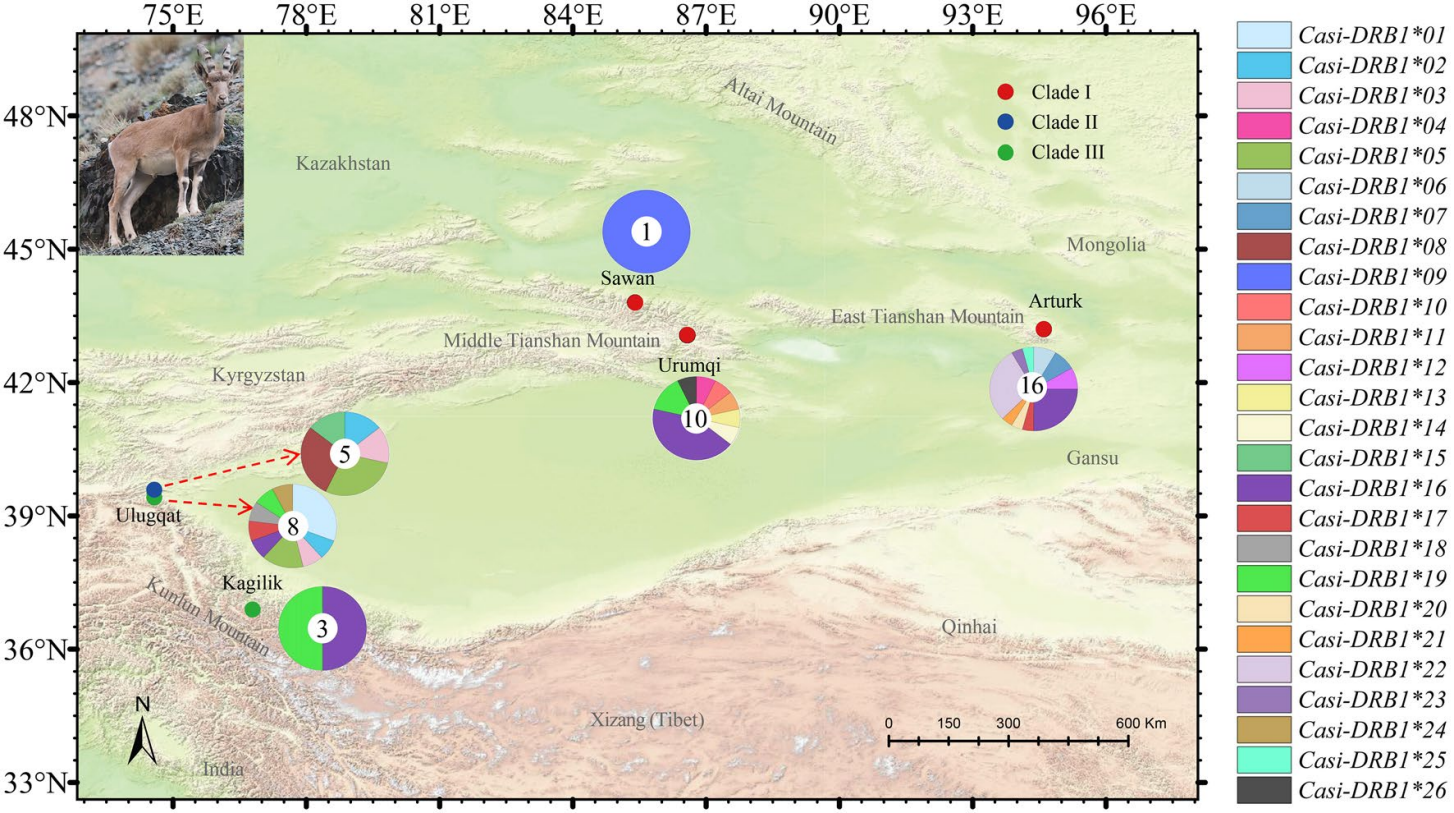
Sampling locations for *Capra sibirica* in Xinjiang, China, in this study. Each small circle on the map indicates sampling locality and different colors signify different clades determined based on the mtDNA analyses^39^. The pie chart shows alleles frequencies (number of alleles) for geographic populations/clades, with each allele in a different color (key at the right). This figure was produced using ArcGIS Pro 2.5.0 (https://www.esri.com/).

A total of 43 samples, including 33 feces, 5 muscle, 4 skin, and 1 liver sample, were analysed. Of these, 10 samples collected from Urumqi, 16 from Arturk, 1 from Sawan, 13 from Ulugqat, and 3 from Kagilik (Fig. 1). All samples in this study were came from samples of our previous study^39^. Tissue samples either taken from individuals died of natural causes or dead individuals that were poached. Individual identity of fecal samples was established according to Abduriyim et al.^40,41^. All fecal and tissue samples were preserved in 96% ethanol, and skin samples were directly frozen in plastic bags at − 80 °C until use.

### Experimental procedures

The total genomic DNA of fecal samples was extracted using an Omega stool DNA extraction kit (Omega Bio-tec, Georgia, USA), and that of muscle and skin samples was extracted using a Tiangen tissue/blood DNA extraction kit (Tiangen Bio-tec, Beijing, China), following the manufacturer's instructions. After electrophoresis detection, DNA concentration and purity were measured by the Thermo Nanodrop 1000 and stored at 4 °C for later use.

Part of MHC class II *DRB1* exon 2 (260 bp, excluding the primer sequences) was PCR amplified using primer pairs of CapDRB1.1F and CapDRB1.2R^42^, because this segment is the most polymorphic region and includes all ABSs necessary for pathogen recognition^12,43^. A PCR reaction volume contained 40–150 ng of DNA, 5 pmol of each primer, 12.5 μL of Tiangen's 2 TaqPCR Master Mix, and then adjusted to a final volume of 25 μL with RNase-Free double distilled water. The PCR thermal cycling conditions were as follows: pre-denaturation at 94 °C for 4 min, followed by 35 cycles of denaturation at 94 °C for 30 s, annealing at 64 °C for 30 s, extension at 72 °C for 45 s, and final extension at 72 °C for 5 min. The PCR products were verified by 2% agarose gel electrophoresis and green fluorescence dye imaging under ultraviolet irradiation, and those with the expected band size were sent for Sanger sequencing bidirectionally using both the forward and reverse primers (Qingke Biology, Xi-an, China).

PCR products assumed to contain more than one sequences were proceeded to cloning and sequencing for allele isolation. PCR products were recovered using a gel recovery kit (Tiangen, Beijing, China), connected to the PMD™19-T plasmid vector (Takara, Tokyo, Japan), then transformed into *Escherichia coli* (DH5α) receptive cells. For selection of cells with positive plasmids, the bacteria were grown on LB solid medium containing ampicillin, IPTG and X-gal at 37 °C overnight. Bacteria containing plasmids with the target PCR product were screened by blue/white selection and direct-colony PCR amplification using M13 forward and reverse primers with the same PCR condition as described earlier. At least 8 clones per sample were bidirectionally sequenced for each individual.

### MHC genotyping

All nucleotide sequences obtained were aligned using MEGA v.6.0^44^. The unique and same sequences were screened using DnaSP v.5.10.01^45^. The final sequences were identified as potentially genuine *DRB1*exon 2 sequences if they matched in the forward and reverse directions, and were detected at least twice in one individual (two independent PCR reactions for one individual) or once each from at least two individuals^46^. Single, unique sequences were omitted, as they may have been PCR chimeras or due to other PCR errors^5,43^. We verified candidate sequences with BLAST searches^47^ at the National Center for Biotechnology Information (NCBI) GenBank database. Final verified sequences were named by consulting the conventions of Klein et al.^48^, and Ballingall and Todd^49^.

### Data analyses

The nucleotide, amino acid, super type diversity, and pairwise population fixation indices (*F*_ST_) of *DRB1* exon 2 for different populations were calculated by DnaSP v.5.10.01^45^, and the neutral selection was analyzed. MEGA v.6.0 were used to estimate the ratio *ω* (*d*_*N*_/*d*_*S*_) of non-synonymous (*d*_*N*_) to synonymous (*d*_*S*_) substitution rates^50^; this ratio provides a measure of selective pressure at the level of individual sites^51^. Values of *ω* > 1 indicate positive selection, while *ω* = 1 and *ω* < 1 indicate neutral evolution and purifying selection, respectively. Values of *d*_*N*_, *d*_*S*_ and *ω* were calculated separately for presumed ABSs deduced according to Reche and Reinherz^52^, non-ABSs, and all sites. HyPhy^53^ implemented in MEGA was used to detect signs of positive selection. In order to examine positive selection across all sites based on maximum likelihood methods, CodeML in PAML 4.9^54^ was employed as well. The likelihood ratio tests (LRTs) were used to compare the four models: M1a, almost neutral; M2a, positive selection; M7, beta; and M8, beta and *ω*, and decide which model best fit our data^51,55,56^. Using LRTs, two nested models (M1a vs. M2a; M7 vs. M8) were compared. Using Bayes Empirical Bayes inference^57^, positively selected locations were found. In addition, using Datamonkey v.2.0^58^, a web-based server for the HyPhy Package, a mixed-effects model of evolution, MEME^59^ analysis was carried out to find codons that had been subject to positive selection.

Gene recombination analysis of *DRB1* exon 2 sequences was performed in RDP4^60^. Specific methods were first used in RDP^61^, GENECONV^62^, MaxChi^63^ and Bootscan^64^, which use default Settings to detect recombination events using Bonferroni correction for multiple comparisons. Recombination events detected by at least three of these methods were then rechecked using all RDP methods available^61^. In addition, we also use the GARD^65^, provided by the Datamonkey webserver^66^, to detect the signatures of recombination breakpoints. In order to avoid the impact of possible gene replication, conversion, and recombination on phylogenetic analysis, we chose Splitstree4 v.4.14.5 to construct a neighbor network of *DRB1* sequences^5,67^.

## Results

### Diversity of *DRB1* alleles

**Figure 2:**
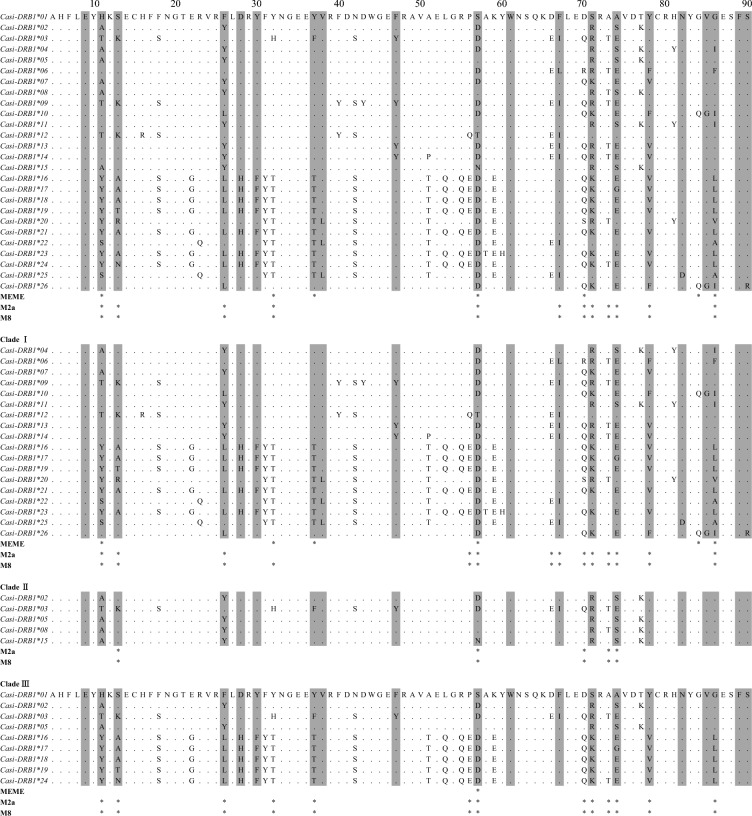
Alignment of deduced amino acid sequences encoded by exon 2 of MHC class II *DRB1* alleles in *Capra sibirica*. Numbers above the amino acid sequences indicate positions in the β1-domain of the DR protein β-chain. Dots indicate amino acids identical to those in Casi-DRB1*01. Putative ABSs as determined by Reche & Reinherz^52^ are shaded. * signs at the bottom of the table indicate sites inferred to be under positive selection by MEME analysis and Bayes Empirical Bayes inference (BEB) using PAML. For the M2a and M8 models in BEB, only significant results are indicated by + (*P* > 95%). Clade II did not find positive selection sites by MEME analysis and is therefore not shown.

Our analytical sequences were 260 bp in length, encode 86 amino acids including 20 ABSs, accounting for 91% of the *DRB1* β1 domain (Fig. 2). We identified 26 presumably functional alleles (PFA) in a total of 43 individuals belong to three mtDNA clades^39^. None of these sequences were pseudogenes. The number of PFA found in a single individual ranged from 1 to 3, indicating existence of 1 or 2 loci per haploid genome in the Siberian ibex. 26 individuals possess only one PFA, implying that these individuals were homozygous. 12 individuals with two PFAs were highly likely heterozygous individuals. Only five individuals had three PFLs (Table A1). Summarizing, most of the individuals in Siberian ibex Xinjiang populations had one *DRB1* locus and could be homozygous.

The most common PFA was Casi-DRB1*16, occurred in 17 individuals that belong to Clade I (12 individuals) and Clade III (5 individuals). The PFAs Casi-DRB1*22 and *19* came after, but we found only in seven and five individuals, respectively. Casi-DRB1*16 and *17* were shared by Clades I and III, Casi-DRB1*02, *03*, and *05* by Clades II and III. Besides, Casi-DRB1*08 and *15* were specific to Clade II, while Casi-DRB1*01, *18*, and *24* were only found in Clade III, and the remaining ones were exclusively occupied by Clade I (Fig. 1, Table A1).

Diversity indices showed a high level of genetic diversity at the nucleotide, amino acid and supertype sequence level for all clades. By comparison, the nucleotide diversity of individuals from Clade I and Clade III was similar, and both were higher than that from CladeII. In terms of amino acid and super type, Clade II had an overall lower level of diversity than Clade I and Clade III as well (Table 1). Tajima’s *D* values were positive, except for Clade II, though none of these values were significant (Table 1). Altogether, this indicates that the MHC class II *DRB1* locus in different clades was likely at the different the stage of bottleneck or selection pressures.

**Table 1:** Genetic diversity at nucleotide, amino acid and supertype levels and neutrality test on the MHC *DRB1* gene for three clades of *Capra sibirica* in Xinjiang, China.

Clade	Sample	Number	Diversity indices			Tajima's *D*
	Size		π	aa	Supertype	
I	27	18/16/14	0.092	0.190	0.427	0.184
II	5	5/5/4	0.035	0.078	0.228	− 0.793
III	11	9/8/8	0.084	0.177	0.402	0.705
Total	43	26/23/20	0.089	0.179	0.406	0.277

The Number column shows, in order, the number of alleles, the number of amino acids, and the number of supertypes.

We calculated the genetic differentiation by *F*_ST_ values, both including and excluding shared alleles between the three Clades. The *F*_ST_ value between clades I and III was negative (Table 2), indicating that within-population genetic differentiation was higher than between-population genetic differentiation. After excluding the shared alleles, the value was positive but very low, implying that the shared alleles were more divergent than the unique alleles in these clades. When including shared alleles, the *F*_ST_ values between clades I and II, II and III were 0.267 and 0.343, respectively (Table 2), indicating a very high level of differentiation.

**Table 2:** Genetic differentiation (*F*_*ST*_) of the MHC *DRB1* gene among the three clades of *Capra sibirica* in Xinjiang, China. The *F*_*ST*_ Values were calculated including (below the diagonal) and excluding (above the diagonal) shared alleles between clades.

Clade	I	II	III
I		0.364	0.030
II	0.267		0.560
III	− 0.013	0.343	

### Recombination and selection on *DRB1*

It was hardly evident that significant recombination signatures exist in our analyses of the *DRB1* exon 2 sequences of the Siberian ibex. Hence, we used all sequences in the downstream analyses. To evaluate selection pressure, we calculated the *ω* ratio of non-synonymous to synonymous substitution rates for positions in the presumed ABSs, non-ABS codons and all codons for three Clades. The *ω* ratio value for ABS codons in *C. sibirica DRB1* was greater than one. Our result thus indicates that variation at the ABS codons were generated and maintained by positive selection. Comparatively, *ω* value for Clade II was nearly twice of that for clades I and II, indicating that the selection intensity on these clades was different (Table 3). This was in line with the results of PAML and MEME analyses that provide evidence for positive selections at the single codon level (Table 4). Likelihood ratio tests (LRT) showed that the M2a and M8 models provided significantly better fits to our data than models without selection (Table 4). The M2a and M8 models identified 13 and 14 positively selected sites in Clade I, respectively, 13 from each model in Clade III, while only five from each model in Clade II (Fig. 2, Table 4), with most of the sites occurring in presumed ABS codons. Finally, the MEME analysis showed six codons under positive selection in Clade I, only one in Clade III, and none in Clade II (Fig. 2).

**Table 3:** Rates (± standard error) of non-synonymous (*d*_*N*_) and synonymous (*d*_*S*_) substitutions and their ratio (*ω*) for the presumed antigen binding sites (ABS), non-ABSs, and sites overall in the β1-domain of the MHC class II *DRB1* genes for *Capra sibirica* in Xinjiang, China.

Substitution type		Number of codons	Clade I	Clade II	Clade III
*d*_*N*_	ABS	20	1.203 ± 0.209	0.244 ± 0.149	0.690 ± 0.186
	Non-ABS	66	0.288 ± 0.140	0.051 ± 0.084	0.132 ± 0.096
	Overall	86	0.514 ± 0.127	0.101 ± 0.081	0.257 ± 0.100
*d*_*S*_	ABS	20	0.658 ± 0.293	0.087 ± 0.288	0.392 ± 0.298
	Non-ABS	66	0.159 ± 0.170	0.015 ± 0.122	0.068 ± 0.208
	Overall	86	0.293 ± 0.158	0.037 ± 0.144	0.167 ± 0.180
*ω*	ABS	20	1.828	2.805	1.760
	Non-ABS	66	1.811	3.400	1.941
	Overall	86	1.754	2.730	1.539

**Table 4:** The results of codon based positive selection analyses using maximum likelihood models in CodeML for MHC *DRB1* exon 2 sequences from *Capra sibirica*. Positively selected sites (PSS), log-likelihood (lnL), the likelihood ratio test (LRT) and probability (*P*) values were presented.

Clade	Models	lnL	Parameter estimates	PSS	LRT	d.f.	*P* value
I	M1a	− 1010.99	*P*_0_ = 0.860, *P*_1_ = 0.140, *ω*_0_ = 0.041, *ω*_1_ = 1.000		M1a vs M2a	2	< 0.01
	M2a	− 972.69	*P*_0_ = 0.572, *P*_1_ = 0.401, *P*_2_ = 0.026, *ω*_0_ = 0.099, *ω*_1_ = 1.000, *ω*_2_ = 13.181	11, 13, 26, 56, 57, 66, 67, 70, 71, 73, 74, 78, 86			
	M7	− 1014.03	*P* = 0.025, *q* = 0.155		M7 vs M8	2	< 0.01
	M8	− 972.72	*P*_0_ = 0.974, *P* = 0.107, *q* = 0.116, *P*_1_ = 0.026, *ω* = 13.366	11, 13, 26, 32, 56, 57, 66, 67, 70, 71, 73, 74, 78, 86			
II	M1a	− 445.45	*P*_0_ = 0.524, *P*_1_ = 0.476, *ω*_0_ = 0.000, *ω*_1_ = 1.000		M1a vs M2a	2	0.0103
	M2a	− 440.88	*P*_0_ = 0.858, *P*_1_ = 0.000, *P*_2_ = 0.142, *ω*_0_ = 0.000, *ω*_1_ = 1.000, *ω*_2_ = 11.496	13, 57, 70, 73, 74			
	M7	− 446.65	*P* = 1.970, *q* = 0.005		M7 vs M8	2	< 0.01
	M8	− 440.88	*P*_0_ = 0.859, *P* = 0.005, *q* = 2.990, *P*_1_ = 0.142, *ω* = 11.496	13, 57, 70, 73, 74			
III	M1a	− 640.12	*P*_0_ = 0.737, *P*_1_ = 0.263, *ω*_0_ = 0.000, *ω*_1_ = 1.000		M1a vs M2a	2	< 0.01
	M2a	− 622.57	*P*_0_ = 0.963, *P*_1_ = 0.000, *P*_2_ = 0.037, *ω*_0_ = 0.546, *ω*_1_ = 1.000, *ω*_2_ = 18.992	11, 13, 26, 32, 37, 56, 57, 70, 71, 73, 74, 78, 86			
	M7	− 640.26	*P* = 0.005, *q* = 0.012		M7 vs M8	2	< 0.01
	M8	− 622.47	*P*_0_ = 0.969, *P* = 0.008, *q* = 0.005, *P*_1_ = 0.031, *ω* = 20.626	11, 13, 26, 32, 37, 56, 57, 70, 71, 73, 74, 78, 86			
All	M1a	− 1132.10	*P*_0_ = 0.898, *P*_1_ = 0.102, *ω*_0_ = 0.039, *ω*_1_ = 1.000				
	M2a	− 1081.75	*P*_0_ = 0.978, *P*_1_ = 0.000, *P*_2_ = 0.022, *ω*_0_ = 0.468, *ω*_1_ = 1.000, *ω*_2_ = 14.736	11, 13, 26, 32, 57, 67, 70, 71, 73, 74, 78, 86	M1a vs M2a	2	< 0.01
	M7	− 1134.53	*P* = 0.016, *q* = 0.103				
	M8	− 1079.73	*P*_0_ = 0.979, *P* = 0.021, *q* = 0.026, *P*_1_ = 0.021, *ω* = 14.775	11, 13, 26, 32, 57, 67, 70, 71, 73, 74, 78, 86	M7 vs M8	2	< 0.01

*ω* equals *d*_*N*_ to *d*_*S*_ ratio; *P*_n_ is the proportion of amino acids in the *ω*_n_ site class; *P* and *q* are parameters of the beta distribution.

### Phylogeny of *DRB1* alleles

**Figure 3:**
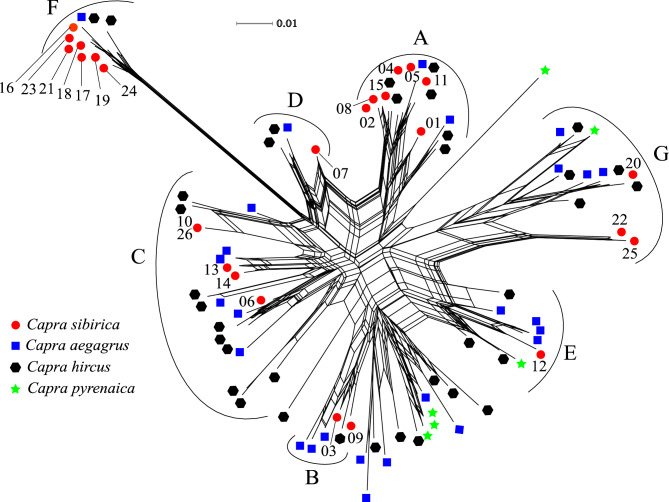
Phylogenetic neighbor network of MHC II *DRB1* exon 2 (233 bp) sequences from *Capra* species, including *C. sibirica* in this study and the remaining three species sequences downloaded from GenBank. The numbers represent allele names with profix Casi-DRB1*.

In the phylogenetic neighbor grid, the *DRB1* exon 2 sequences of *C. sibirica* did not form separate groups according to the geographical population results, but merged into sequences of other species of genus *Capra*, forming groups of A, B, C, D, E, F, and G (Fig. 3). Trans-species polymorphism (TSP) was clearly evident, with some exon 2 sequences from particular *C. sibirica* being more closely related to sequences from other *Capra* species than to those from the same species. The E and G groups are composed of four types of *Capra*: *C. sibirica*, *C. aegagrus*, *C. hircus*, *C. pyrenaica DRB* sequences; The remaining A, B, C, D, and F groups contain only *DRB* sequences of *C. sibirica*, *C. aegagrus*, and *C. hircus*.

## Discussion

In this study, we examined the sequence diversity of the MHC Class II *DRB1* exon 2 of *C. sibirica* from the eastern Tianshan, the middle Tianshan, and the Kunlun Mountains, with a maximum distance of about 2000 km and an average of 4500–5250 m-height mountain peaks^68^, which seriously hindered the genetic exchange between populations^39^. In addition, anthropogenic factors led these populations to a drastic decline in size.

Some may wonder allelic dropout could happen when fecal samples used for genetic studies. Nonetheless, it was reported that the detection of MHC alleles using fecal DNA was generally consistent with the results of blood DNA^69^. Considering the worst case, to prevent the possible allelic dropout, we first used the fresh fecal samples and preserved them in 96% ethanol at − 80 °C to avoid DNA degradation; we then bidirectionally sequenced at least eight (at most 24) independent clones for an individual; and all sequences from all individuals together were used for searching identical nucleotide sequences which were true alleles, as different individuals probably carry same alleles^43,69^. Moreover, the allele numbers obtained from tissue samples were consistent with that from fecal samples (Table A1). These indicate the obtained alleles in our present study were reliable.

### MHC *DRB1* diversity and divergence

Indirect indicators of the immunological fitness of populations, MHC genes are adaptive genetic markers useful in wild animal populations of concern for protection^3,70^. Many species, which went through severe bottlenecks, show very low levels of genetic diversity at the MHC, for example, mountain goats, *Oreamnos americanus*^35^ and Galà pagos penguin, *Spheniscus mendiculus*^36^. Conversely, despite a rinderpest epidemic-induced bottleneck, high allelic diversity for the *DRB3* gene was reported for the African buffalo, *Syncerus caffer*^71^. Our study on MHC class II *DRB1* exon 2 allowed, for the first time, a comparison of genetic variation among *C. sibirica* populations that genetically highly diverged and underwent population reduction in size in Xinjiang, China^18,39^. We found higher allelic diversity of MHC class II *DRB1* loci in *C. sibirica* compared to other congenerics. Although the 26 PFAs we detected in 43 *C. sibirica* individuals (Fig. 1, Table A1) seem to be lower than the 22 PFAs among 25 samples reported for its domestic counterpart from six different breeds^72^, only seven PFAs were found among 132 individuals of *Capra pyrenaica* with two subspecies, *C. p. hispanica* and *C. p. victoria*^73^. This high number of alleles is mainly attributed the pathogens and parasites they suffer from^22,23^, and to some degree to this species’ sexual segregation and preference for different habitats and diets for both genders^74,75^. Though the reduction in size in *C. sibirica* may have an impact on the heterozygosity of the MHC *DRB* locus, since more than half of the studied individuals (26 out of 43 samples) possess a single PFA (Table A1).

Individuals of *C. sibirica* clade II had low levels of diversity at the allelic, nucleotide, amino acid, and supertype levels relative to those of Clades I and III (Table A1, Table 1), indicating that the impact of population declines and/or environmental pathogenic pressures on the different geographic populations was different^76^. We also cannot exclude the possibility that this difference is due to the low number of samples analyzed; thus, dense sampling is needed for further related studies.

Although we did not find a single allele shared by all three *C. sibirica* clades, but found alleles common to two clades. For instance, the alleles Casi-DRB1*16 (the most frequent one identified in individuals from east and middle Tianshan mountains, and Kunlun Mountains), Casi-DRB1*17 and Casi-DRB1*19 were shared by clades III and I, while alleles Casi-DRB1*02 and Casi-DRB1*05 were shared by clades III and II (Fig. 1 and Table A1). The radiation of these clades dates back around 6.75 million years ago^39^, indicating preservation of these alleles in *C. sibirica* for such a long evolutionary time. An allele was conserved in the genus *Meles* for nearly 2 million years^43^; two alleles were even shared by multiple species from different genera in mustelids^77^, diverged more than 11 million years ago^78^; some MHC allele surprisingly preserved among different family^5^. This is probably because *C. sibirica* populations in Xinjiang, China subjected to same pathogenic burdens for a long evolutionary time, as polymorphism in MHC gene was pathogen driven^79^.

Meantime, we found more population- or clade-specific alleles (Fig. 1, Table A1), implying high differentiation at this locus. The long radiation time (3.3–6.7 million years) of these populations or clades probably illuminates this phenomenon^39^. MHC genes also showed genetic differentiation between populations in some mammal species^76,80,81^. The *F*_*ST*_ values between Clade I and Clade II and Clade II and Clade III were greater than 0.25 (Table 2), indicating large genetic differences. What is puzzling is that the *F*_*ST*_ value between the Clade I and Clade III was slightly negative. This is because that the differences within populations were greater than the differences between populations^82^. The negative value of *F*_*ST*_ generally interpreted as 0^83^, which means clade I and III were not differentiated. However, after excluding the shared alleles between these clades in *F*_ST_ estimation, it turns to be low differentiation (Table 2). Overall, this in part supports the pattern of mitochondrial DNA^39^.

The number of PFAs identified for our studied individuals shows 1 or 2 loci of the *DRB* gene (Table A1), with a low frequency of 2 loci (5 out of 43 individuals), though. Many species in the genus *Capra*, including Alpine ibex (*Capra ibex*), Spanish ibex (*Capra pyrenaica*), and Himalayan tahr (*Hemitragus jemlahicus*), have only one locus of *DRB*^84^. It can be seen that the ancestor of *Capra* species is supposed to possess a single locus at the MHC *DRB* gene. Despite the small portion of individuals with two loci, they split into clades I and III, respectively. We assume that the one locus likely emerged from the other locus through gene duplication^6^. Even if no evidence supports the occurrence of recombination events due likely to the shortness of our analytical sequences, intergenic recombination or gene conversion may explain this phenomenon as well^85^, and they might happen twice independently in these two clades. A population genome study on the MHC class II region will help us demonstrate this notion.

### Evolution of the *DRB1* gene

Generally, MHC gene polymorphism were generated and retained by gene recombination^84,86^, gene duplication^6^, balancing selection^79,87^, and/or positive selection^43,79,87^. In our study, we did not find any significant signature of recombination events, convincing us that gene recombination was not the reason for generation of MHC diversity. Nonetheless, we found more rare alleles than shared or high frequency alleles (Table A1). This is suggestion of balanced polymorphisms include negative-frequency-dependent selection, where rare alleles are favored. Besides, we also found a notable excess of nonsynonymous over synonymous substitutions at ABSs, in different clades (Table 3). In our phylogenetic relationship analysis, *DRB1* sequences of *C. sibirica* were grouped with the sequences of its counterparts (Fig. 3), suggesting that some alleles are phylogenetically more closely related to the alleles of other species than to those of its own, a typical trans-species polymorphism^88^, which is reported for MHC genes of many species^5,43,79,87,89^. All of these were the evidence supporting the presence of long-term balancing selection in the *C. sibirica*, considering that the *Capra* species were diverged approximately 6 million years ago^39^. Moreover, the PAML CodeML and MEME analyses identified up to 12 positively selected sites, most of which coincide with the ABSs (Fig. 2 and Table 4), suggested that the sequence variation of *DRB1* genes was driven by positive selection due to pathogenic burdens^19–23^. In sum, our results together indicate that selection was the main force shaping and maintaining *DRB1* gene polymorphism in *C. sibirica*.

It is worthy to mention that we as well as observed an exceeded nonsynonymous relative to synonymous substitutions at the none-ABSs in all clades of *C. sibirica* (Table 3), which is in line with the positive selection analyses that showed several positively selected sites out of ABSs (Fig. 2 and Table 4). This is consistent with the results of Abduriyim et al.^43^ in a species of Canidae. Considering that all MHC studies deduce the ABS locations based on human MHC structure^52^, the actual location of ABSs in the MHC Class II DR β-chain of *C. sibirica*, radiation from humans took place as far back as 95 million years^90^, may be different. This leaves an open question if ABSs of MHC molecule in all mammals were overlapped.

## Conclusions

Despite the level of genetic diversity in clade II is lower than that in other clades, and thus requires close attention in future conservation plans, the overall diversity (i.e., allelic, nucleotide, amino acid and supertype diversity) of MHC class II *DRB1* genes in *C. sibirica* Xinjiang populations after a bottleneck have not rapidly been lost. The differential preference for habitat and food of two sexes might contribute to generation and retain of MHC diversity. The genetic differentiation of clades/populations was to some extent in support of the results by Wang et al.^39^ on mtDNA. The diversity of MHC *DRB1* genes in *C. sibirica* was shaped and maintained by selection, both positive and balancing selection.

### Supplementary Information

Supplementary Table 1.

## Data Availability

The sequences we obtained have been deposited in the NCBI databases under accession numbers OR257668–OR257693.
