# Supplementary material for: Diversity and evolution of the MHC class II DRB gene in the Capra sibirica experienced a demographic fluctuation in China
Source: Sci Rep. 2023 Nov 7;13:19352. doi: 10.1038/s41598-023-46717-5 (PMC10630338; doi:10.1038/s41598-023-46717-5)
Supplement: Supplementary file 1 — Supplementary Table 1. [file 41598_2023_46717_MOESM1_ESM.pdf]

Diversity and Evolution of the MHC Class II *DRB* Gene in an alpine ungulate, the Siberian ibex (*Capra sibirica*), experienced a demographic fluctuation in China

Pei-Pei Dong [1#], Rui-Rui Wang[1#], Shamshidin Abduriyim[1, 2*]

1 College of Life Science, Shihezi University, Shihezi 832003, Xinjiang, China

2 Xinjiang Production and Construction Corps Key Laboratory of Oasis Town and Mountain-Basin System Ecology, Shihezi University, Shihezi 832003, Xinjiang, China

# These authors contributed equally to this work.

* Correspondence to **Shamshidin Abduriyim**

Email: shamshidin@shzu.edu.cn

Appendix

Table A1 Distribution and frequency of MHC class II *DRB1* alleles among 43 *Capra sibirica* individuals in Xinjiang, China. The clades I to III were determined based on mitochondrial sequence analyses[32].

No.	Clade	location	Type	Individual identity	*Casi-DRB1**																										Number of verified alleles
					1	2	3	4	5	6	7	8	9	*10*	11	12	13	14	15	**16**	17	**18**	19	20	**21**	22	**23**	24	25	*26*	
1	I	Arturk	Liver	CSYW7																+											1
2		Arturk	Feces	CSYWa						+	+													+							3
3		Arturk	Feces	CSYWb																+	+				+						3
4		Arturk	Feces	CSYWc																						+					1
5		Arturk	Feces	CSYWd												+															1
6		Arturk	Feces	CSYWe												+															1
7		Arturk	Feces	CSYWi																+											1
8		Arturk	Feces	CSYWj																+							+				2
9		Arturk	Feces	CSYWk																+											1
10		Arturk	Feces	CSYWB																						+			+		2
11		Arturk	Feces	CSYWD						+																+					2
12		Arturk	Feces	CSYWE							+									+											2
13		Arturk	Feces	CSYWF																						+					1
14		Arturk	Feces	CSYWX1																						+					1
15		Arturk	Feces	CSYWX2																						+					1
16		Arturk	Feces	CSYWX3																						+					1
17		Sawan	Skin	SLG									+																		1
18		Urumqi	Feces	CShx1													+	+													2
19		Urumqi	Feces	CShx2																+			+								2
20		Urumqi	Feces	CShx6																+											1

21		Urumqi	Feces	CShx10				**+**												+											2
22		Urumqi	Feces	CShx11										**+**																	1
23		Urumqi	Feces	CShx13											**+**																1
24		Urumqi	Feces	CShx14																										**+**	1
25		Urumqi	Feces	CShx16																+											1
26		Urumqi	Feces	CShx19																+											1
27		Urumqi	Feces	CShx20																+			+								2
28	II	Ulugqat	Feces	CSnj1								**+**																			1
29		Ulugqat	Feces	CSnj3		+																									1
30		Ulugqat	Skin	CSnjP1			+																								1
31		Ulugqat	Skin	CSnjP2					+										**+**												2
32		Ulugqat	Skin	CSnjP3					+			**+**																			2
33	III	Ulugqat	Feces	CSnj5	**+**															+	+										3
34		Ulugqat	Feces	CSnj6																								**+**			1
35		Ulugqat	Feces	CSnj8					+																						1
36		Ulugqat	Feces	CSnj9		+														+			+								3
37		Ulugqat	Feces	CSnj10					+																						1
38		Ulugqat	Feces	CSnj11	**+**																	**+**									2
39		Ulugqat	Muscle	CSnjJ1	**+**		+													+											3
40		Ulugqat	Muscle	CSnjJ2	**+**																										1
41		Kagilik	Muscle	CSYC1																+											1
42		Kagilik	Muscle	CSYC2																			+								1
43		Kagilik	Muscle	CSYC3																+			+								2
				Total	4	2	2	1	4	2	2	2	1	1	1	2	1	1	1	17	2	1	5	1	1	7	1	1	1	1	65

The bolded + marks represent alleles unique to that clade. The bolded and italic allele names signify identical amino acid sequences. The allele names with same color mean identical supertype sequences (ABS sequences).
